# Insights regarding sirtuin-dependent gene regulation during white koji production

**DOI:** 10.1080/19420889.2022.2051844

**Published:** 2022-03-16

**Authors:** Taiki Futagami, Masatoshi Goto

**Affiliations:** a Education and Research Center for Fermentation Studies, Faculty of Agriculture, Kagoshima University, Kagoshima, Japan; b United Graduate School of Agricultural Sciences, Kagoshima University, Kagoshima, Japan; c Department of Applied Biochemistry and Food Science, Faculty of Agriculture, Saga University, Saga, Japan

**Keywords:** *Aspergillus luchuensis* mut. *kawachii*, white koji, gene regulation, NAD^+^-dependent histone deacetylase, sirtuin

## Abstract

White koji, a solid-state culture of *Aspergillus luchuensis* mut. *kawachii* using grains such as rice and barley, is used as a source of amylolytic enzymes and citric acid for the production of shochu, a traditional Japanese distilled spirit. We previously characterized changes in gene expression that affect the properties of white koji during the shochu production process; however, the underlying regulatory mechanisms were not determined. We then characterized the NAD^+^-dependent histone deacetylase sirtuin, an epigenetic regulator of various biological phenomena, in *A. l*. mut. *kawachii* and found that sirtuin SirD is involved in expression of α-amylase activity and citric acid accumulation. In this addendum study, we measured the NAD^+^/NADH redox state and found that the NAD^+^ level and NAD^+^/NADH ratio decrease during koji production, indicating that sirtuin activity declines in the late stages of koji culture. By comparing these results with transcriptomic data obtained in our previous studies, we estimate that approximately 35% of the gene expression changes during white koji production are SirD dependent. This study provides clues to the mechanism of gene expression regulation in *A. l*. mut. *kawachii* during the production of white koji.

Koji fungi are used in the production of traditional Japanese fermentation products such as sake, miso, and soy source [1]. Koji fungi are grown in solid-state culture on steamed grains such as rice and barley and play a crucial role in supplying amylolytic enzymes such as α-amylase and glucoamylase, which degrade the starch contained in source grains. The white koji fungus *Aspergillus luchuensis* mut. *kawachii* is commonly used in the production of shochu, a traditional distilled spirit in Japan [2]. In addition to amylolytic enzymes, *A. l*. mut. *kawachii* produces a large amount of citric acid, which prevents contamination with undesired microbes during the fermentation process.

During the production of white koji, the cultivation temperature is gradually increased to 40°C and then reduced to 30°C [2] (Figure 1a). The latter process of lowering the temperate is performed to enhance the accumulation of citric acid in koji [3]. In a previous study, we investigated the effect of temperature on global gene expression in *A. l*. mut. *kawachii* during the barley koji production process to characterize the gene expression changes that control citric acid productivity [4]. Citric acid is generated as an intermediate of the tricarboxylic acid (TCA) cycle, which is downstream of glycolysis. Based on DNA microarray data, it was concluded that cultivation at 40°C is thermally stressful for *A. l*. mut. *kawachii* and that heat adaptation leads to reduced citric acid production via the activation of pathways that branch from glycolysis [4]. Expression of genes involved in the trehalose shunt, glycerol synthesis, and pentose phosphate pathways, which also function downstream of glycolysis, was downregulated after lowering the cultivation temperature from 40 to 30°C. Thus, metabolic flux to the TCA cycle seems to be enhanced by lowering the culture temperature to accumulate citric acid. However, the regulatory mechanisms underlying these gene expression changes have not been elucidated.

**Figure 1.**
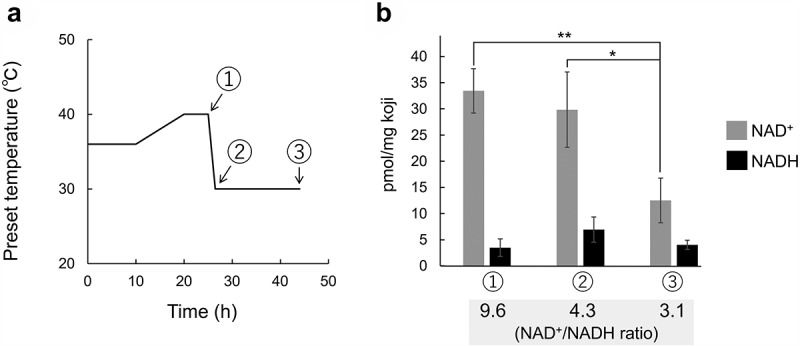
Experimental conditions and NAD^+^/NADH redox state in production of white koji. (a) Cultivation temperature. The temperature was increased from 36 to 40°C over a 25-h period and then decreased to 30°C [4]. Circled numbers (1, 2, and 3) indicate sampling points. (b) Concentrations of NAD^+^ and NADH and NAD^+^/NADH ratio at the three sampling points during barley koji production. Means and standard deviations were determined from the results of three independent experiments. Welch’s *t*-test: **, *p* < 0.01; *, *p* < 0.05.

To identify the regulator of gene expression changes associated with the quality of white koji, we recently characterized five sirtuin homologous genes (*sirA, sirB, sirC, sirD*, and *sirE*) in *A. l*. mut. *kawachii* [5]. Sirtuin is an NAD^+^-dependent class III histone deacetylase that functions as an epigenetic regulator by controlling histone acetylation levels [^6–8^]. In the genus *Aspergillus*, sirtuin regulates the expression of genes involved in a variety of processes, such as secondary metabolite production, enzyme production, and maintenance of cell wall integrity [^9–13^]. We found that disruption of *sirD* leads to reduced levels of acid-stable α-amylase activity and citric acid production in white koji, accompanied by dynamic gene expression changes [5]. For instance, the expression of genes related to the trehalose shunt, pentose phosphate pathway, and citric acid export process is downregulated in the *sirD* disruptant. It should also be noted that the acid-stable α-amylase encoding gene (*asaA*) was downregulated by *sirD* disruption. This may explain the reduced level of acid-stable α-amylase activity.

In this study, we evaluated the role of SirD in regulating the gene expression changes during the white koji production process. Because the NAD^+^ level is known to control the activity of sirtuin [9^,^^12^^,14–16^], we measured the NAD^+^ and NADH concentrations in barley koji and calculated the NAD^+^/NADH ratio at three sampling points, as shown in Figure 1a. Barley koji was prepared as described in our previous study [4], and 15 grains of barley koji were mechanically broken using a multibead shocker instrument (3,000 rpm, 15s; Yasui Kikai, Osaka, Japan). Levels of NAD^+^ and NADH in the crushed koji samples were determined using a NAD/NADH Quantitation Colorimetric kit (BioVision, Milpitas, CA) and an iMark microplate reader (Bio-Rad, Hercules, CA) according to the manufacturers’ protocols. The concentration of NAD^+^ was significantly lower in the latter half of the koji production process (Figure 1b). The NAD^+^ level at sampling point 3 was 37% of the level at sampling point 1 and 42% of the level at sampling point 2. In addition, the NAD^+^/NADH ratio declined from sampling points 1 to 3. These results suggest that the NAD^+^-dependent activity of sirtuin is downregulated by lowering the culture temperature during koji production. It was also observed that the total amount of NAD^+^ and NADH was declined from sampling points 2 to 3 (Figure 1b). The cellular levels of NAD^+^ and NADH are known to be controlled by *de novo* and salvage synthesis and degradation. For example, the nudix hydrolase hydrolyzes NAD^+^ and NADH and decreases the cellular NAD^+^ and NADH in *A. nidulans* [9,16,17].

Next, we performed a comparative transcriptomic analysis under two different conditions that affect citric acid productivity: changes in SirD-dependent gene expression and different cultivation temperatures during koji production (Figure 2). Following *sirD* disruption in *A. l*. mut. *kawachii*, we identified significant changes in the expression of 2,908 genes, which included 1,318 upregulated genes and 1,590 downregulated genes using cap analysis of gene expression (Gene Expression Omnibus [GEO] accession number GSE132729) [5]. In addition, using DNA microarray analysis (GEO accession number GSE58454), we identified significant changes in the expression of 1,114 genes (including 566 upregulated genes and 548 downregulated genes) in *A. l*. mut. *kawachii* after lowering the cultivation temperature [4]. Log_2_-fold changes of less than −0.5 or greater than 0.5 were considered significant in both analyses [4,5]. The comparative analysis revealed that among the genes differentially regulated by *sirD* disruption, a large number were either up- or downregulated by lowering the temperature (Figure 2). Among the 566 genes upregulated by lowering the temperature, 168 overlapped with genes upregulated by *sirD* disruption (left-hand side of Figure 2). In addition, among the 548 genes downregulated by lowering the temperature, 214 overlapped with genes downregulated by *sirD* disruption (right-hand side of Figure 2). These data are consistent with our hypothesis that SirD activity declines during the latter half of the koji production process based on the NAD^+^/NADH redox state (Figure 1b) and suggest that approximately 35% of the genes that are up- or downregulated by lowering the temperature are regulated via a SirD-dependent pathway. Importantly, however, these results are not consistent with the observations that *sirD* disruption reduces citric acid productivity while lowering the temperature enhances citric acid productivity. This discrepancy might be associated with the incomplete overlap in differentially regulated citric acid production–related genes between *sirD* disruption and lowering the culture temperature. For example, we previously identified significant changes in the expression of 27 genes, which included genes involved in glycolysis and gluconeogenesis, pentose phosphate pathway, trehalose synthesis and hydrolysis, glycerol metabolism, TCA cycle, glyoxylate shunt, γ-aminobutyrate shunt, and citrate transport process in the *sirD* disruptant (Locus tags are shown in Figure 2) [5]. Twenty-three of the 27 genes were downregulated by *sirD* disruption (right-hand side of Figure 2), indicating that these genes may be positively regulated by SirD. However, only 10 of the 23 genes were overlapped with genes downregulated by lowering the cultivation temperature.

**Figure 2.**
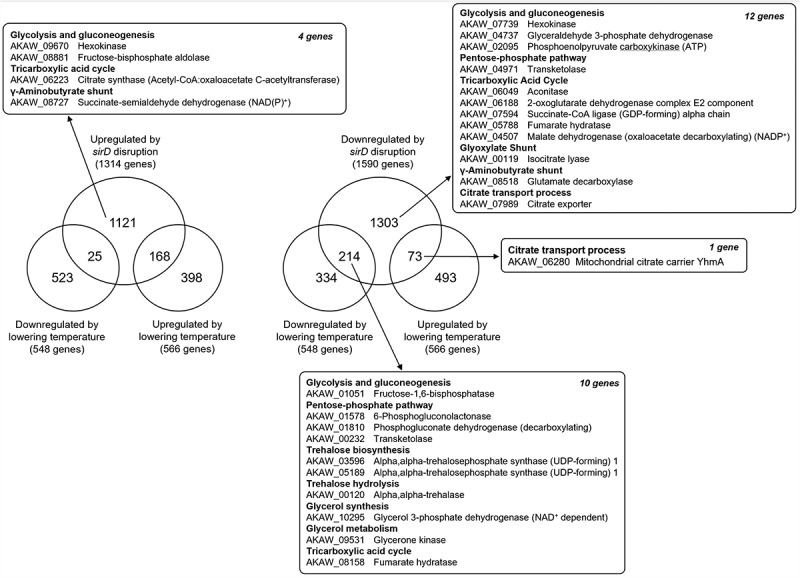
Venn diagrams showing the number of genes identified by comparative transcriptomic analyses. Genes regulated by SirD (*sirD*-disruptant versus control and lowering the temperature [as shown in Figure 1a]) were compared. Transcriptomic data were obtained from the Gene Expression Omnibus (accession numbers GSE58454 and GSE132729) [4,5].

In *A. nidulans*, SirE (an ortholog of *A. l*. mut. *kawachii* SirD) is believed to regulate the activity of primary and secondary metabolic processes that mediate the transition from the exponential to stationary growth phase [12]. During barley koji production, the higher temperature (40°C) induces stress in *A. l*. mut. *kawachii*, whereas lowering the temperature to 30°C enhances the activation of central metabolism pathways, including glycolysis and the TCA cycle [4]. Thus, lowering the culture temperature might enhance growth during the transition from exponential to stationary growth phase. Further studies of SirD-dependent gene regulation could expand our understanding of the mechanisms by which gene expression is regulated during koji culture and facilitate optimization of the fermentation process.

## Data Availability

The datasets analyzed during the current study are available in the Gene Expression Omnibus (accession numbers GSE58454 and GSE132729) [4, 5].
